# Does ICD-11 exclude the harm caused due to second hand and third hand smoke? - A critique

**DOI:** 10.1016/j.lansea.2022.100075

**Published:** 2022-09-20

**Authors:** Yatan Pal Singh Balhara, Siddharth Sarkar

**Affiliations:** National Drug Dependence Treatment Center and Department of Psychiatry, All India Institute of Medical Sciences (AIIMS), New Delhi, India

The most recent version of International Classification of Diseases (ICD-11) has made significant changes to the disorders related to use of psychoactive substances.^1^ Besides inclusion of newer diagnostic categories, amendments have been made to some of the existing categories. Episode of harmful use of substance and harmful pattern of use of substances are such examples. While the former is a new introduction in the ICD-11, the later has been revised to be more inclusive.

One of the most significant amendments to both these diagnostic categories is inclusion of ‘harm to others’ in addition to ‘harm to self’ because of use of the substance. This means that unlike previously where the evidence to the harm of physical or mental health of the user was required to make the diagnosis of ‘harmful use’, the ICD-11 has recognized harm to the physical and mental health of others a sufficient requirement to diagnose single episode, as well as harmful pattern of use of a substance.

The category of “mental and behavioral disorders due to use of tobacco” in ICD-10 has now been replaced with the category ‘disorders due to use of nicotine’ in ICD-11. However, unlike other substances such as alcohol and opioids, the provision of ‘harm to others’ is not listed in the description section of the episode of harmful use of nicotine and harmful pattern of use of nicotine.^2^ The section on diagnostic requirement has listed the provision to ‘harms to others’ like other substances but mentions that ‘such behavior is unlikely to arise from nicotine intoxication’. In ICD-11, the harm to others includes any form of physical harm, including trauma, or mental disorder that is directly attributable to behaviour due to intoxication due to use of a substance. The use of tobacco products (the main product forms in which nicotine is consumed) exposes others to the second-hand smoke (SHS) and third hand smoke (THS) both under the state of intoxication (mentioned in ICD-11 as a less likely event), and use without accompanying intoxication. Given this, ICD-11 does not capture the harm caused by SHS and THS.

The WHO has identified that worldwide, SHS causes an estimated 600,000 premature deaths a year, the majority (64%) among women.^3^ According to the CDC, “second-hand smoke causes stroke, lung cancer, and coronary heart disease in adults. Children who are exposed to second-hand smoke are at increased risk for sudden infant death syndrome, acute respiratory infections, middle ear disease, more severe asthma, respiratory symptoms, and slowed lung growth”.^4^ Non-smokers who are exposed to SHS at home or at work increase their risk of developing lung cancer by 20–30%.^5^ The list of the adverse physical health effects of SHS is even longer and includes eye irritation, headaches, nasal symptoms, cough, and allergic attacks.

Similarly, a publication on policy implications of the THS reported it to be a significant public health concern.^6^ It reported that studies have found THS in cars, homes, and hotel rooms that ban indoor/in-car smoking. Also, children in smoking households have 5–7 times more nicotine exposure than those from non-smoking households despite of in-house smoking bans. An estimated 3 million children younger than 6 years of age are estimated to be exposed to SHS or THS more than three days a week.^6^

Despite this body of evidence, the harm to the health of others due to exposure to SHS and THS has not been captured by the ICD -11 conceptualization of ‘harm to others’ and has not been included in any other category as well. In fact, of all the psychoactive substances the evidence of harm to the physical health of others is most compelling for tobacco. While concerns have been expressed about the quality and quantum of evidence for harm to the health of others due to different psychoactive substance,^1^ the evidence in this context for nicotine is definitive. Another concern about the introduction of the concept of harm to others in certain diagnostic categories in ICD-11 is about concerns over how to quantify and measure the harm. The same may be a concern in context of nicotine as well, as the amount of harm caused to the health of others by use of nicotine by one person on a particular occasion may be difficult to quantify with complete certainty. However, the adverse effects of the SHS and THS are well established and are a result of cumulative exposure over time. In addition, certain other adverse physical health effects such as eye irritation, headaches, nasal symptoms, cough, and allergic attacks can result from even a single episode of exposure to SHS or THS.

In keeping with the inclusion of harm to others in certain diagnoses related to use of psychoactive substances in ICD-11, there is need to ensure that the public health consequences of SHS and THS do not go uncaptured.

## Contributors

YPSB conceptualized the article. Both YPSB and SS wrote the manuscript, edited it, and approved the final version.

## Declaration of interests

None.
